# Sentinel Lymph Node Biopsy for Early-Stage Oral Cavity Cancer: Analysis of Diagnostic Accuracy and False-Negative Cases

**DOI:** 10.3390/jcm15072545

**Published:** 2026-03-26

**Authors:** Rodrigo Lozano-Rosado, Alvaro De-Bonilla-Damia, Guiomar Martin-Lozano, Alberto Garcia-Perla-Garcia, Jose-Luis Gutierrez-Perez, Pedro Infante-Cossio

**Affiliations:** 1 Department of Oral and Maxillofacial Surgery, Virgen del Rocio University Hospital, 41013 Seville, Spain; drlozanomaxilofacial@gmail.com (R.L.-R.); gml_03@hotmail.com (G.M.-L.); agarciaperla@us.es (A.G.-P.-G.); jlgp@us.es (J.-L.G.-P.); 2 Department of Nuclear Medicine, Virgen del Rocio University Hospital, Instituto de Biomedicina (IBIS), 41013 Seville, Spain; abonilladamia@gmail.com; 3 Department of Surgery, School of Medicine, University of Seville, 41009 Seville, Spain; 4 Department of Stomatology, School of Dentistry, University of Seville, 41009 Seville, Spain

**Keywords:** oral cancer, T1–T2 N0, sentinel lymph node biopsy, false negatives, cervical metastasis, neck dissection

## Abstract

**Background/Objectives**: Identifying the causes of sentinel lymph node biopsy (SLNB) failure in early-stage oral cavity squamous cell carcinoma (OCSCC) is essential for refining surgical protocols and optimizing patient selection. This study aimed to evaluate the diagnostic performance, predictors of false-negative (FN) results, and long-term oncological outcomes of SLNB in patients with early-stage OCSCC. **Methods**: A retrospective, single-centre cohort study was conducted on 220 patients with cT1–cT2 N0 M0 OCSCC who were surgically treated between 2017 and 2024. Preoperative lymphatic mapping was performed using ^99m^Tc-nanocolloid and SPECT/CT. All sentinel lymph nodes (SLNs) underwent an ultrastaging protocol involving serial sectioning and immunohistochemistry. Diagnostic accuracy, survival outcomes, and clinicopathological predictors of FNs were analysed. **Results**: The SLN identification rate was 99.1%. Metastatic involvement was detected in 49 patients (22.3%), preventing 77.7% of the cohort from undergoing unnecessary neck dissection. Bilateral lymphatic drainage was observed in 55.9% of floor of the mouth tumours. During a median follow-up of 36 months, the diagnostic performance showed a sensitivity of 81.7%, a negative predictive value of 93.6%, and an overall accuracy of 95.0%. Analysis of the 11 FN cases showed that both depth of invasion (DOI) (6.0 mm vs. 3.0 mm; *p* = 0.010) and maximal tumour dimension (25 mm vs. 15 mm; *p* = 0.0008) were significant predictors of diagnostic failure. The five-year overall survival rate was significantly superior in patients with negative SLNs compared to the SLN-positive group (82% vs. 61%; *p* < 0.001). **Conclusions**: SLNB is an accurate and reliable staging tool for early-stage OCSCC, providing personalised lymphatic mapping that harmonizes oncological efficacy with the avoidance of overtreatment. However, an increased DOI and a larger tumour size significantly raise the risk of FN events, indicating the need for close postoperative surveillance in these high-risk subgroups.

## 1. Introduction

Oral cavity squamous cell carcinoma (OCSCC) poses a significant global health challenge, with locoregional control being a critical factor in determining survival rates [1,2]. Approximately half of all OCSCC cases in developed countries are diagnosed at an early stage (Stages I and II) [3]. Cervical lymph node metastases constitute the most critical individual prognostic factor, as it reduces overall survival (OS) by approximately 50% [4,5,6]. Even in cT1–cT2 N0 disease, up to one-third of patients develop occult nodal metastases that are undetectable using contemporary imaging modalities [7,8]. Consequently, the optimal management of clinically negative neck (cN0) remains one of the most debated topics in head and neck oncology.

Historically, elective neck dissection (END) has served as the gold standard for early-stage (cT1–cT2) OCSCC, given its documented efficacy in enhancing regional control, OS, and disease-specific survival (DSS) [9,10]. Nevertheless, this strategy results in overtreatment for approximately 70–80% of patients who undergo a potentially unnecessary invasive procedure, exposing them to postoperative complications such as shoulder dysfunction, chronic pain, and aesthetic sequelae [11]. To mitigate overtreatment and the postoperative morbidity associated with END, sentinel lymph node biopsy (SLNB) has emerged as a validated, minimally invasive alternative [12,13]. Initially established in melanoma and breast cancer, SLNB has demonstrated comparable oncological efficacy in oral cancer through multiple multicentre trials and recent meta-analyses, offering accurate staging and survival outcomes equivalent to END while significantly reducing treatment-related morbidity [14,15,16].

While SLNB demonstrates high detection rates in cN0 necks, notable heterogeneity persists regarding its availability, implementation, and international application criteria [2,17,18]. Consequently, the National Comprehensive Cancer Network (NCCN) guidelines advocate for either SLNB or END as equivalent modalities [19,20]. Assessing the long-term efficacy of SLNB within routine clinical settings—beyond the constraints of controlled trials—remains a fundamental priority, as is the identification of factors underlying diagnostic failures. Elucidating the mechanisms leading to false-negative (FN) results is crucial for refining surgical techniques and optimizing patient selection criteria.

This study presents a comprehensive, eight-year, single-centre experience with SLNB for early-stage OCSCC. Our primary objective was to evaluate the diagnostic accuracy of the technique and conduct a detailed analysis of FN cases to identify patterns of failure and enhance oncological safety within the context of real-world clinical practice at a tertiary referral centre.

## 2. Materials and Methods

### 2.1. Study Design and Ethical Approval

A retrospective, single-centre cohort study was performed at the Department of Oral and Maxillofacial Surgery of the Virgen del Rocio University Hospital (Seville, Spain), spanning an eight-year period (2017–2024). The study protocol was approved by the local Clinical Research Ethics Committee and was conducted in accordance with the principles of the Declaration of Helsinki. The manuscript was prepared in compliance with the Strengthening the Reporting of Observational Studies in Epidemiology (STROBE) guidelines.

### 2.2. Patient Selection

Patients with a histologically confirmed diagnosis of OCSCC at clinical stages cT1–cT2 N0 M0 who underwent surgical treatment were consecutively included. Preoperative clinical staging was based on physical examination and imaging modalities, comprising contrast-enhanced computed tomography (CT) or magnetic resonance imaging (MRI), in accordance with the 8th edition of the American Joint Committee on Cancer (AJCC) Cancer Staging Manual [21].

- Inclusion criteria: Primary cT1 or cT2 tumours with a clinically negative neck (cN0) as assessed by palpation and imaging; surgical treatment indication approved by the hospital’s Multidisciplinary Head and Neck Oncology Board; and written informed consent for the SLNB procedure.
- Exclusion criteria: Evidence of cervical or distant metastases at presentation; recurrent disease; previous history of neck dissection (ND) or cervical radiotherapy; synchronous tumours; cases where transoral resection was not feasible via a standard surgical approach; or patients deemed unfit for surgery due to severe comorbidities.

### 2.3. Lymphoscintigraphy Protocol and SLNB Procedure

Within 24 h prior to surgery, 3 mCi (111 MBq) of ^99m^Tc-albumin nanocolloid was administered via four peritumoural submucosal injections. The imaging protocol integrated planar lymphoscintigraphy with SPECT/CT. When feasible, dynamic planar imaging was performed immediately following radiotracer injection to identify early drainage patterns. Subsequently, 300 s static acquisitions (anterior and lateral projections) were obtained at 10 min and 2 h post-injection. SPECT/CT was performed after the delayed (2 h) planar images to provide precise three-dimensional anatomical localisation. Criteria for SLN identification on preoperative imaging included the visualisation of afferent lymphatic channels, timing of appearance, identification of the lymphatic basin, and the relative intensity of radiotracer uptake. The cutaneous projection of each SLN was marked on the patient’s skin with indelible ink to facilitate intraoperative guidance.

Surgical procedures commenced with the resection of the primary tumour to mitigate the shine-through effect while ensuring negative surgical margins. Intraoperative localisation of SLNs was achieved using a handheld gamma probe within the pre-marked cervical areas. Ex vivo radioactivity measurements were routinely performed to validate in vivo detection, with radioactive counts recorded over 10 s intervals. Following the extirpation of all nodes identified via SPECT/CT or intraoperative probe detection, the surgical bed was systematically scanned. The procedure was considered complete only when the residual radioactivity in the surgical field was <10% of the counts recorded in the most radioactive SLN.

### 2.4. Histopathological Processing

A standardised nodal ultrastaging protocol was applied to maximise diagnostic sensitivity. All retrieved sentinel lymph nodes (SLNs) were fixed in formalin and paraffin-embedded. Processing involved 3 μm thick serial sections at 50 μm intervals; initial sections were evaluated via conventional haematoxylin and eosin (H&E) staining. For specimens that were negative on routine H&E assessment in three consecutive sections, immunohistochemical analysis was performed using a pancytokeratin AE1/AE3 antibody cocktail. Metastatic deposits were categorised based on the AJCC 8th edition criteria [21]: macrometastases (>2 mm), micrometastases (0.2–2 mm), and isolated tumour cells (ITCs, <0.2 mm).

### 2.5. Follow-Up and Definition of False Negatives

Patients with SLN involvement (macrometastases or micrometastases) underwent therapeutic completion ND (typically involving cervical levels I–III/IV or modified radical dissection), based on the hospital’s Multidisciplinary Head and Neck Oncology Board consensus. Conversely, patients with negative SLNs or ITCs were managed with a rigorous surveillance protocol, consisting of clinical and radiological evaluations bimonthly during the first two years and quarterly thereafter, for a minimum follow-up period of five years. Management of involved or close surgical margins of the primary tumour was tailored to each case, encompassing options such as surgical re-resection to achieve wider margins, adjuvant radiotherapy (with or without chemotherapy), or intensified clinical observation.

An FN was defined as a histopathologically negative SLNB followed by the development of regional recurrence (ipsilateral or contralateral nodal metastasis) in the presence of local control at the primary tumour site. Contralateral recurrences were classified as FN regardless of the initial lymphatic drainage pattern, representing a failure of the procedure to map the true basin or detect subclinical metastatic disease. Consequently, the FN cohort encompassed all patients with initially negative nodes (pN0) or ITCs who subsequently experienced regional failure. ITCs were initially classified as pN0 in accordance with AJCC criteria [21]; however, any patients with ITCs who subsequently developed cervical metastases were included in the FN cohort to avoid underestimating diagnostic failure. Diagnostic confirmation of these events was established through the integration of the Neck Imaging Reporting and Data System (NI-RADS) radiological assessment and ultrasound-guided fine needle aspiration (US-guided FNA). To investigate these diagnostic failures, a comprehensive audit was performed for all FN cases, involving a systematic re-evaluation of medical records, preoperative imaging studies, and histopathological sections.

### 2.6. Variables and Statistical Analysis

Baseline demographic characteristics (age and sex) and clinicopathological parameters of the primary tumour were recorded, including anatomical subsite, tumour thickness, depth of invasion (DOI), perineural invasion (PNI), surgical margins, and treatment modalities. Tumour size was defined as the maximal tumour dimension, whereas DOI was measured according to the AJCC 8th edition criteria [21], extending from the basement membrane of the adjacent epithelium to the deepest point of tumour invasion. Regarding the SLN status, data were collected on the results of the ultrastaging protocol, the size of the largest metastatic deposit (macrometastasis vs. micrometastasis), the involved nodal basins, laterality of lymphatic drainage, and the presence of extranodal extension (ENE). The primary endpoints were the SLN identification rate, histopathological status (positive or negative), negative predictive value (NPV), and overall diagnostic accuracy. Secondary endpoints included the false-negative (FN) rate and long-term survival outcomes (OS and DSS) stratified by SLN status.

Statistical analyses were performed using IBM SPSS Statistics software, version 26.0 (IBM Corp., Armonk, NY, USA). Continuous variables are presented as means (±standard deviation, SD) or medians (interquartile range, IQR), depending on data distribution. Comparisons between continuous variables were carried out using the Mann–Whitney U test, whereas categorical variables were analysed using Fisher’s exact test or Pearson’s chi-square test, as appropriate. Diagnostic performance metrics—including sensitivity, specificity, NPV, and accuracy—were calculated using 2 × 2 contingency tables and are reported with confidence intervals (95% CI). OS, DSS, and disease-free survival (DFS) were estimated using the Kaplan–Meier method and plotted as cumulative survival curves including numbers-at-risk tables. Survival outcomes were analysed according to the initial histopathological status of the SLN. Inter-group comparisons were performed using the log-rank test. All statistical tests were two-tailed, and a *p*-value < 0.05 was considered statistically significant.

## 3. Results

### 3.1. Patient Characteristics, Treatment, and Follow-Up

Initially, a total of 222 consecutive patients with cT1–cT2 N0 OCSCC were screened for the study. The SLN identification rate was 99.1% (220/222). In two cases (0.9%), unsuccessful preoperative lymphatic mapping occurred; these patients were consequently excluded, resulting in a final study cohort of 220 patients. Definitive histopathological assessment of the SLNs identified metastatic disease in 49 patients (22.3%), who were thus upstaged to pN+.

Baseline demographic and clinicopathological characteristics, stratified by SLN status, are summarised in Table 1. The mean patient age was 63.7 years (range: 26–87 years), with a balanced sex distribution (50.9% male, 49.1% female). Although all cases were initially staged as cT1–cT2, histopathological evaluation resulted in the upstaging of 23 tumours (10.5%) to pT3, based on the DOI ≥10 mm criterion introduced in the 8th edition of the AJCC staging system [21]. The median maximal tumour dimension was 17 mm (IQR: 12–22 mm), and in 201 patients (91.3%), the DOI was ≤9 mm. Negative surgical margins were achieved in 182 cases (82.7%), and PNI was identified in 20 cases (9.1%). A median of three SLNs was excised per patient (IQR: 2–4).

Univariate analysis revealed that a higher probability of SLN positivity was significantly associated with an advanced pathological T stage (*p* = 0.0003), greater tumour thickness (*p* = 0.0002), deeper DOI (*p* = 0.0009), and the presence of PNI (*p* = 0.045).

Surgical and follow-up data are summarised in Table 2. Surgery alone was the treatment modality in 62.7% of patients, whereas 20.5% received adjuvant radiotherapy and 16.8% underwent concurrent chemoradiotherapy. ND was performed in 55 patients (25.0%), of whom 47 underwent the procedure following a positive SLN finding. The median interval between SLNB and completion ND was 22 days (IQR: 19–30). 58 patients were diagnosed with additional malignancies: 20.5% developed extraoral primary tumours and 5.9% developed second primary tumours of the oral cavity. The median follow-up duration for the entire cohort was 36 months (IQR: 17.6–63.0).

### 3.2. Primary Tumour Location and Sentinel Lymph Node Distribution

Table 3 summarizes the anatomical distribution of the primary tumours and the topographical localisation of the identified SLNs across cervical levels. The most frequent primary subsite was the lateral border of the tongue (54.1%), followed by the floor of the mouth (15.5%). Cervical level II was the predominant nodal drainage basin in both ipsilateral and contralateral patterns. Additionally, bilateral lymphatic drainage was observed in 20.2% of tumours located at the lateral border of the tongue and 55.9% of tumours arising from the floor of the mouth.

### 3.3. Sentinel Lymph Node Histopathological Status

Among the 49 patients (22.3%) upstaged to pN+, the majority (37 cases; 75.5%) presented with a single metastatic SLN, while 11 (22.4%) and one (2.1%) patient showed involvement of two and three SLNs, respectively (Table 4). Regarding nodal laterality, three patients exhibited isolated contralateral SLN positivity, and two presented with bilateral metastatic involvement. ENE was identified in 6.1% of positive SLNs. The metastatic burden within the SLNs was categorized as macrometastasis (>2 mm) in 39 patients (79.6%) and micrometastasis (0.2–2 mm) in 10 patients (20.4%). Following subsequent completion ND in SLN-positive cases, additional metastases in non-sentinel lymph nodes (NSLNs) were detected in 16 patients (32.6%), with ENE present in 18.4% of these cases.

### 3.4. Diagnostic Accuracy and False-Negative Analysis

A total of 11 FN cases were identified, all of which manifested within the first 12 months of follow-up. Contingency table analysis identified 49 true-positive (TP) cases (patients with a positive SLN), 11 FN cases (patients with a negative SLN who subsequently developed regional recurrence), and 160 true-negative (TN) cases (patients with a negative SLN who remained free of regional disease at the end of follow-up).

Accordingly, the diagnostic performance of SLNB in our cohort yielded the following metrics:

- Sensitivity: 81.7% (95% CI: 69.5–90.9).
- NPV: 93.6% (95% CI: 88.8–96.9).
- FN rate: 18.3% (95% CI: 9.1–30.5).
- Overall diagnostic accuracy: 95.0% (95% CI: 92.1–97.9).

The clinicopathological characteristics of the 11 FN cases are summarized in Table 5. The clinical profile revealed a median age of 61 years, with a marked predominance of lingual tumours (91%). Regarding primary tumour metrics, the median maximal dimension was 25 mm, the median DOI was 6 mm, and the median surgical margin width was 2.5 mm. PNI was present in 18% of these cases. In terms of lymphatic mapping, a median of three SLNs was retrieved per patient, and contralateral drainage was identified in 36% of the FN subgroup. The median time to the development of regional recurrence was 5 months (range: 1–10 months). Regarding the management of diagnostic failures, salvage ND was a viable option in only three of the 11 FN cases, all of whom survived and remain free of disease with a median follow-up of 24 months (range: 12–62 months) after the salvage surgery. The remaining eight patients presented with unresectable disease—complicated by distant pulmonary metastases in three cases—and were managed via radiotherapy, chemotherapy, immunotherapy, or a combination thereof. Ultimately, five patients (45.5%) were successfully salvaged, including all three individuals who underwent surgery.

A comparative analysis between the FN (n = 11) and TN (n = 160) subgroups is detailed in Table 6. Univariate analysis identified that tumour size was significantly larger in the FN group compared to the TN group (25 mm vs. 15 mm; *p* = 0.0008). Similarly, the DOI was significantly deeper in the FN subgroup than in the TN subgroup (6.0 mm vs. 3.0 mm; *p* = 0.0102). Conversely, no statistically significant differences were detected regarding age, the prevalence of PNI, surgical margin width, the total number of SLNs retrieved, or the presence of contralateral SLN involvement.

### 3.5. Survival Analysis

The overall mortality rate was 20.0% (n = 44). Specifically, 27 deaths (15.8%) occurred among the 171 patients with a negative SLN, compared to 17 deaths (34.7%) in the 49 patients with a positive SLN. Disease-related mortality—directly attributed to the biological progression of OCSCC—accounted for 70.4% (n = 31) of all deaths. Regarding disease recurrence, local failure was detected in 19 patients (8.6%), regional metastasis in 20 (9.1%), and synchronous locoregional recurrence in 19 (8.6%).

**Figure 1:**
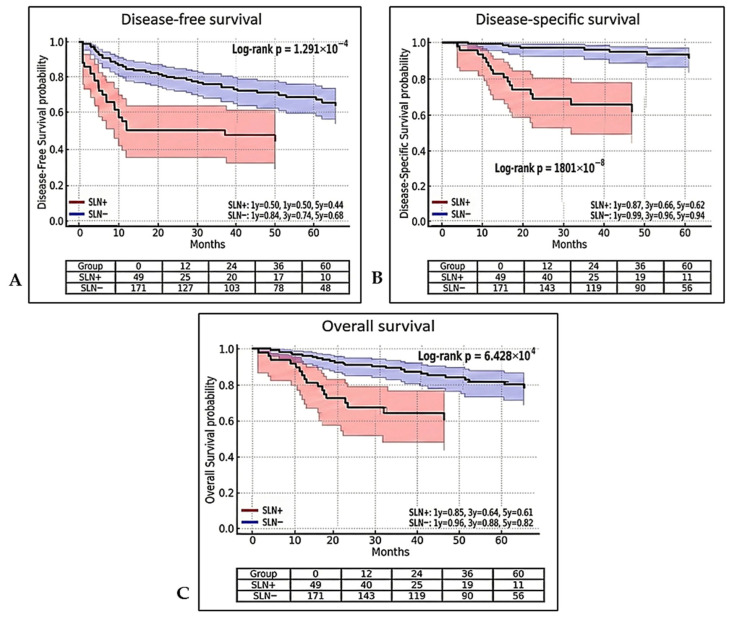
Kaplan–Meier survival curves stratified according to SLN pathological status (SLN-positive vs. SLN-negative). The plots display the probabilities of (**A**) DFS, (**B**) DSS, (**C**) OS. The blue areas represent the SLN-negative subgroup and the red areas, the SLN-positive subgroup. Shaded areas surrounding the main survival lines indicate the 95% CI. One-, three-, and five-year survival rates are provided for each group. Inter-group statistical significance was evaluated using the log-rank test, with corresponding *p*-values displayed in each panel. The risk tables indicate the number of patients at risk at specific follow-up intervals (months).

SLN positivity was identified as a highly significant prognostic determinant for long-term outcomes, as Kaplan–Meier analysis revealed significant differences across all survival endpoints (Figure 1). The estimated 5-year DFS was 68% in the SLN-negative group versus 44% in the SLN-positive group (*p* < 0.0001). Similarly, DSS and OS were significantly superior in patients with a negative SLN (94% vs. 62%; *p* < 0.000001, and 82% vs. 61%; *p* < 0.0006, respectively).

## 4. Discussion

Over the last two decades, the management of cN0 neck in early-stage OCSCC has progressively evolved towards less invasive strategies. The landmark randomized trial by D’Cruz et al. [10] solidified END as the reference standard by demonstrating a clear survival advantage over observation. However, that study also underscored the clinical imperative to identify alternative approaches—such as SLNB—capable of achieving comparable oncological outcomes while mitigating the morbidity associated with overtreatment. Within this framework, SLNB has emerged as a validated staging technique, offering a diagnostic performance equivalent to END with a significantly more favourable safety profile [2].

Derived from an eight-year institutional experience involving 220 patients, our results corroborate the technical feasibility and oncological safety of SLNB documented in numerous prospective multicentre trials [11,17,22,23]. Consistent with NCCN 2024 guidelines [20], our study reinforces SLNB as a robust alternative to END for cT1–cT2 N0 OCSCC in experienced centres, providing equivalent survival outcomes while significantly mitigating the postoperative morbidity associated with more invasive neck procedures [2,15,17,24,25]. In our cohort, unnecessary ND—and its associated complications—was avoided in 77.7% of patients. Notably, SLNB enabled the detection of unexpected contralateral or bilateral lymphatic drainage in 10% of cases; such drainage patterns would have remained unaddressed by standard ipsilateral END [26]. Moreover, our identification of DOI and maximal tumour dimension as predictors of diagnostic failure complements current recommendations, highlighting high-risk subgroups that warrant cautious interpretation of negative results and intensified postoperative surveillance. These results emphasize the clinical utility of SLNB as a personalized lymphatic mapping strategy beyond conventional neck management. Consequently, our data consolidated this technique as a cornerstone of our institutional protocol for early-stage OCSCC.

The 99.1% SLN identification rate observed in our series is highly consistent with results reported in landmark multicentre trials, such as the SENT study (99.5%) [17]. This confirms the technical reproducibility of the procedure within the routine clinical workflow of a tertiary referral centre, extending its validity beyond the strictly controlled environment of clinical trials. Such a high identification rate highlights the consistency of our multidisciplinary protocol, which integrates the systematic use of SPECT/CT imaging and a specific surgical sequence: prioritizing primary tumour resection before SLN localization [19,23]. This strategy is particularly critical for mitigating the shine-through effect [22,27], especially in challenging anatomical subsites like the floor of the mouth. In these cases, the proximity of the radiotracer at the primary tumour bed may obscure radioactive signals from level I and II cervical lymph nodes. While recent technical refinements—such as targeted radiotracers like ^99m^Tc-tilmanocept or adjunctive indocyanine green fluorescence—show promise [24], our results demonstrate that the optimization of established nanocolloid-based protocols remains a highly reliable standard for SLNB.

The clinicopathological profile of our cohort underscores the biological heterogeneity inherent in early-stage OCSCC. Although the study population was confined to tumours staged clinically as cT1–cT2, 10.5% were histopathologically upstaged to pT3 due to a DOI ≥ 10 mm, in accordance with the AJCC 8th edition criteria [21]. This prevalence of clinical understaging highlights the prognostic significance of DOI, not only as a primary factor for pathological T-classification but also as a key predictor of occult nodal metastasis. However, the recent literature has emphasized the inherent limitations of relying on DOI as a standalone preoperative metric. Because an accurate DOI assessment is fundamentally contingent upon definitive histopathological examination following resection, current evidence cautions against using it as an isolated determinant for surgical decision-making in the preoperative phase [28,29,30].

As expected, our results exhibited that SLN positivity was significantly associated with a more advanced pathological T stage, greater tumour thickness, a deeper DOI, and the presence of PNI. However, occult metastases were also observed in categories traditionally considered low-risk, indicating that no single clinicopathological parameter can completely exclude subclinical nodal involvement in early-stage OCSCC. In our series, PNI emerged as a robust predictor of occult disease—a feature traditionally linked to impaired regional control and diminished DFS. Similarly, increased tumour thickness does not merely reflect a higher biological tumour burden; it likely signifies alterations within the tumour microenvironment that facilitate a phenotypic shift toward a more invasive and aggressive growth pattern. Collectively, our data suggest that tumour thickness, DOI, and PNI should not be viewed as isolated variables, but rather as synergistic manifestations of a neoplasm with high metastatic potential. Consequently, the identification of these high-risk features in the surgical specimen—even in early-stage disease with clear surgical margins of resection—should be interpreted as a clinical indication to intensify postoperative surveillance of the cN0 neck.

Regarding therapeutic management, surgery alone was the definitive treatment for 62.7% of our patients, successfully maintaining a minimally invasive approach to the neck through the SLNB-based strategy. A formal assessment of morbidity was purposely excluded from our analysis, as the functional superiority of SLNB over END—specifically regarding shoulder dysfunction and surgical site complications—is already strongly documented in the literature [15]. Adjuvant therapy (radiotherapy or concurrent chemoradiotherapy) was strictly reserved for patients with confirmed nodal metastasis or high-risk features in the primary tumour, such as positive margins or ENE. Notably, additional malignancies were identified in 31.8% of the cohort (26.8% extraoral primary tumours and 5.0% second primary oral tumours). This finding aligns with the field cancerization theory, reinforcing the notion that despite the excellent regional control provided by SLNB, these patients require close and prolonged surveillance. In this regard, our median follow-up of 36 months ensures that the study period effectively covers the critical window during which the vast majority of regional recurrences and second primary oral tumours typically manifest.

The topographical analysis of lymphatic drainage patterns highlights the inherent variability and complexity of the lymphatic network in OCSCC. In our series, the predominance of tumours involving the lateral border of the tongue (54.1%) and the floor of the mouth (15.5%) correlated with cervical level II as the preferential SLN nodal basin, in both ipsilateral and contralateral drainage patterns. A finding of significant clinical impact was the substantial prevalence of bilateral lymphatic drainage, observed in 55.9% of floor of the mouth tumours and 20.2% of lateral tongue tumours. These data indicate that a standard unilateral END would have been oncologically inadequate for more than half of the patients with floor of the mouth carcinomas. Consequently, SLNB functions not only as a diagnostic staging procedure but as a dynamic functional mapping modality. It is capable of identifying cross-over drainage pathways that transcend the anatomical rigidity of conventional ND schemes, thereby ensuring a truly individualized and comprehensive regional management strategy [26].

The detection of micrometastases in 20.4% of the pN+ cases represents a vital diagnostic finding. These results reinforce the capacity of SLNB to identify low-volume occult metastatic disease with greater precision than conventional assessment. Indeed, such deposits would likely have remained undetected during the routine histopathological examination of large END specimens. This enhanced diagnostic yield is directly attributable to the ultrastaging protocol, which incorporates serial sectioning and immunohistochemistry. This methodology enables a meticulous and exhaustive nodal evaluation that would be neither logistically feasible nor economically sustainable if applied to the dozens of lymph nodes typically retrieved in a comprehensive ND specimen.

The detection of metastases in NSLNs in 32.6% of patients who underwent completion ND confirms that while the SLN is frequently the sole site of nodal involvement (75.5%), its positivity serves as a high-fidelity indicator of metastatic risk throughout the remaining cervical levels. A finding of significant prognostic weight was the incidence of ENE. While identified in only 6.1% of positive SLNs, the incidence of ENE increased substantially to 18.4% within the NSLNs retrieved in completion ND specimens. This coexistence of micrometastases and foci of ENE in clinically cN0 patients illustrates the biological heterogeneity of lymphatic dissemination and reinforces the role of SLNB in identifying candidates who require therapeutic ND and adjuvant therapy.

Our findings regarding NSLN involvement are highly congruent with previously published data [30]. In the European multicentre SENT trial [17], NSLN involvement was reported in 20–30% of pN+ patients. Similarly, Garrel et al. and Hasegawa et al. [15,16] documented residual tumour burden in the remaining neck ranging from 25% to 34%. The consistency of our results with these series reinforces the accuracy of our protocol. By identifying additional nodal disease in nearly one-third of pN+ patients, these data indicate that SLN positivity is not an isolated event but a reliable biological marker of the metastatic potential of the primary tumour, thereby justifying completion ND to ensure definitive regional control. Taken together, these findings suggest that, with the oncological effectiveness of SLNB now firmly established, the current challenge lies in defining precise selection criteria and ensuring its standardized implementation in routine surgical practice.

The diagnostic performance achieved in our series—characterized by a sensitivity of 81.7% and an NPV of 93.6%—positions SLNB as a highly reliable modality for early-stage OCSCC. Our 18.3% FN rate aligns with the approximately 82% global sensitivity reported in the meta-analysis by Suárez-Ajuria et al. [30]. Variations across the literature likely stem from heterogeneity in tumour subsite distribution—specifically the complex lymphatic drainage of the tongue and floor of the mouth—and differences in ultrastaging protocols. Furthermore, our adoption of a strict definition of diagnostic failure, which encompasses both ipsilateral and contralateral recurrences, combined with prolonged follow-up, prevents the underestimation of events often seen in studies with shorter surveillance periods. Nevertheless, a focused analysis of the 11 FN cases revealed a distinct high-risk clinical profile. These failures were predominantly associated with a greater local tumour burden, manifested by larger maximal dimensions (25 mm) and a deeper median DOI (6 mm). Notably, 91% of FN events occurred in tongue tumours, with 36% of these cases exhibiting aberrant contralateral drainage patterns. A clinically significant observation was the early manifestation of these diagnostic failures; the median time to regional recurrence was only 5 months, and all FN events occurred within the first postoperative year. This temporal pattern suggests that micrometastatic disease was likely already present and potentially bypassing the SLN or overwhelming the detection threshold of the current ultrastaging protocol at the time of the surgery.

Univariate comparison between the FN and TN subgroups confirmed that maximal tumour dimension (25 mm vs. 15 mm) and DOI (6.0 mm vs. 3.0 mm) were significantly greater in cases of diagnostic failure. These findings underscore the hypothesis that a high local tumour burden is the primary factor underlying undetected regional recurrence. Notably, the absence of significant differences in variables such as the number of SLNs retrieved or the frequency of contralateral drainage suggests that FN events are unlikely to be attributable to technical shortcomings or surgical omissions. Instead, these failures are likely driven by the inherent biological behaviour and infiltrative capacity of the primary tumour. Consequently, while the 95.0% overall diagnostic accuracy supports the routine implementation of SLNB, a negative result—particularly in larger tumours with an aggressive growth pattern—should be interpreted with clinical vigilance and may warrant intensified postoperative monitoring.

It should be noted that, although FN events are documented throughout the literature, they are frequently addressed as secondary endpoints, with relatively few studies offering a detailed characterization of their specific clinical nuances [11,17,31]. The alignment of our FN rate with established benchmarks reinforces the hypothesis that these occurrences do not merely represent isolated procedural oversights but rather reflect an intrinsic biological threshold of lymphatic staging in OCSCC. In this context, our study contributes valuable granular data by characterizing FN events within a large, well-defined cohort, thereby contextualising their true impact on overall oncological safety. Consistent with previous reports, FN cases in our series were infrequent and did not preclude successful regional control; the implementation of close postoperative surveillance facilitated early detection and effective salvage treatment [13]. Collectively, these findings advocate for a paradigm shift: SLNB results should be interpreted within a comprehensive oncological framework rather than as a binary indicator of diagnostic success or failure.

The prognostic weight of histopathological SLN status is clearly illustrated by the Kaplan–Meier survival analysis. Our findings demonstrate that SLN positivity transcends its role as a mere indicator of regional disease extent; it serves as a pivotal determinant of both DSS and OS. Furthermore, the survival trajectories observed in our cohort are highly consistent with previous reference studies established for END in comparable populations with early-stage lesions [17,23]. The marked divergence between the survival curves of SLN-positive and SLN-negative patients highlights that the detection of occult metastatic disease identifies a biological subgroup characterized by intrinsically aggressive tumour behaviour. Crucially, despite the systematic implementation of completion ND and tailored adjuvant therapy in pN+ cases, this subgroup continued to exhibit significantly inferior survival outcomes. This suggests that regional lymph node involvement, even at the microscopic level, remains a prognostic challenge that reflects the underlying metastatic potential of the disease.

Several limitations of the present study warrant consideration. First, its retrospective and single-centre design may introduce inherent selection bias and potentially limit the generalizability of the findings to other clinical settings [32]. However, the substantial cohort size—considerably larger than most published institutional series on SLNB for early-stage OCSCC [24,33,34]—and the strict adherence to a standardized multidisciplinary protocol mitigate this risk and ensure high technical consistency. Second, while the median follow-up of 36 months is sufficient to capture the vast majority of regional recurrences (all of which occurred within the first postoperative year in our series), it barely exceeds the critical threshold of long-term stability time. Third, the two-stage surgical approach could theoretically delay the initiation of adjuvant therapy in pN+ patients; nonetheless, current evidence suggests that this staged management does not result in clinically meaningful delays or adverse prognostic impacts [35]. Finally, the relatively low frequency of FN cases limited the statistical power to perform a robust multivariate analysis, as such modelling would be prone to overfitting and erroneous inferences due to insufficient statistical power [36,37]. Despite this constraint, the translational value of this study lies in the identification of tangible clinical indicators—specifically maximal tumour dimension and DOI—that provide clinically relevant information for identifying high-risk patients in routine clinical practice.

The primary objective of this study was not a head-to-head comparison between surgical modalities, but rather an evaluation of SLNB outcomes within the framework of real-world clinical practice. In this regard, as established by de Bree et al. [38], longitudinal clinical follow-up of the untreated neck serves as a more reliable reference standard for validating SLNB accuracy than histopathological examination alone. This is because any occult disease overlooked during staging will inevitably manifest as regional recurrence over time, whereas routine pathological processing of END specimens frequently lacks the sensitivity to detect low-volume micrometastatic disease. Thus, the patient’s clinical progress is the ultimate measure of diagnostic success. Nevertheless, further multicentre studies are essential to better elucidate the pathophysiological mechanisms underlying FN events and to bolster the external validity of these results across diverse clinical environments.

## 5. Conclusions

In conclusion, our eight-year experience supports SLNB as a reliable and oncologically safe alternative to END for cT1–cT2 N0 OCSCC. By yielding a diagnostic accuracy of 95.0% and an NPV of 93.6%, this technique effectively spared 77.7% of patients from the morbidity of unnecessary surgery while providing personalised lymphatic mapping that could identify aberrant lymphatic pathways in 10.0% of cases. Nevertheless, increased DOI and larger tumour size significantly raise the risk of FN results, requiring cautious interpretation and close surveillance in these subgroups. The marked divergence in survival rates between SLN-positive and SLN-negative patients highlights the strength of SLNB as a prognostic factor and its ability to balance oncological efficacy with avoiding overtreatment.

## Figures and Tables

**Table 1:** Clinical and histopathological characteristics according to SLN involvement (n = 220).

Characteristic		Total (n = 220)	Negative SLN (n = 171)	Positive SLN (n = 49)	*p*-Value *
Age, mean ± SD	Years	63.7 ± 12.7	63.2 ± 12.8	65.7 ± 12.5	0.142
Sex, n (%)	Male	112 (50.9%)	86 (50.3%)	26 (53.1%)	0.913
	Female	108 (49.1%)	85 (49.7%)	23 (46.9%)	
	pT1	134 (60.9%)	116 (67.8%)	18 (36.7%)	0.0003
Pathological stage T (pT), n (%)	pT2	63 (28.6%)	42 (24.6%)	21 (42.9%)	
	pT3 re-staged	23 (10.5%)	13 (7.6%)	10 (20.4%)	
Tumour thickness, median (IQR) mm		17 (12–22)	15 (10–20)	20 (17–25)	0.0002
Depth of invasion, n (%)	<3 mm	71 (32.3%)	65 (38.0%)	6 (12.2%)	0.0009
	3–9 mm	130 (59.1%)	97 (56.7%)	33 (67.3%)	
	≥10 mm	19 (8.6%)	9 (5.3%)	10 (20.4%)	
Surgical margin, n (%)	Affected	38 (17.3%)	26 (15.2%)	12 (24.5%)	0.430
	1–4 mm	85 (38.6%)	67 (39.2%)	18 (36.7%)	
	≥5 mm	97 (44.1%)	78 (45.6%)	19 (38.8%)	
Perineural invasion, n (%)		20 (9.1%)	10 (5.8%)	10 (20.4%)	0.045
SLN per patient, median (IQR)		3 (2–4)	3 (2–4)	3 (2–4)	0.440

* *p*-values obtained with the Mann–Whitney U test for age, tumour thickness, and SLN per patient; Pearson’s chi-square test was applied to all remaining categorical comparisons.

**Table 2:** Surgical and follow-up data (n = 220).

		Total (n = 220)	Negative SLN (n = 171)	Positive SLN (n = 49)
Treatment modality, n (%)	Surgery alone	138 (62.7%)	138 (80.7%)	0 (0.0%)
	Surgery + radiotherapy	45 (20.5%)	18 (10.5%)	27 (55.1%)
	Surgery + radio/chemotherapy	37 (16.8%)	15 (8.8%)	22 (44.9%)
Additional malignancies, n (%)	Extraoral primary tumour	45 (20.5%)	35 (20.5%)	10 (20.4%)
	Second primary oral tumour	13 (5.9%)	10 (5.8%)	3 (6.1%)
Follow-up (months), median (IQR)		36 (17.6–63)	39.9 (20.7–65)	22.5 (12–52)

**Table 3:** Location of the primary tumour and distribution of SLN by cervical levels.

		Ipsilateral SLN (n) by Cervical Level					Contralateral SLN (n) by Cervical Level					Bilateral SLN n (%)
Primary Subsite	Total, n (%)	I	II	III	IV	V	I	II	III	IV	V	
Lateral tongue	119 (54.1%)	53	186	93	20	17	8	16	8	-	-	24 (20.2%)
Floor of mouth	34 (15.5%)	32	25	8	7	7	8	10	3	3	3	19 (55.9%)
Ventral tongue	19 (8.6%)	13	24	8	-	3	1	6	2	-	-	6 (46.2%)
Lower alveolar	14 (6.4%)	6	9	2	-	2	4	3	-	-	1	5 (35.7%)
Buccal mucosa	8 (3.6%)	7	6	-	-	1	-	-	-	-	-	-
Retromolar	5 (2.3%)	6	5	-	-	-	-	-	-	-	-	-
Dorsum of tongue	4 (1.8%)	-	8	3	1	1	1	3	2	-	-	3 (75%)
Upper alveolar	4 (1.8%)	2	5	1	-	-	-	1	-	-	-	1 (25%)
Base of tongue	3 (1.4%)	-	4	1	-	-	-	-	-	-	-	-
Uvula	3 (1.4%)	-	6	-	-	1	-	4	-	1	-	3 (100%)
Lip	3 (1.4%)	8	5	-	-	-	1	1	-	-	-	2 (66.7%)
Tip of tongue	2 (0.9%)	2	1	1	2	-	2	1	-	-	-	2 (100%)
Soft palate	2 (0.9%)	-	4	-	-	-	-	1	-	-	-	1 (50%)

Cervical levels I–V refer to standard neck nodal levels: Level I (submental/submandibular), level II (upper jugular), level III (mid-jugular), level IV (lower jugular), and level V (posterior triangle).

**Table 4:** Histopathological findings in positive SLN cases.

	Histopathological Finding	n (%)
Positive SLN per patient, n (%)	1 SLN	37 (75.5%)
	2 SLNs	11 (22.4%)
	3 SLNs	1 (2.1%)
Location of SLN positivity, n (%)	Ipsilateral SLN	44 (89.8%)
	Contralateral SLN	3 (6.1%)
	Bilateral SLN	2 (4.1%)
Extranodal extension in SLN, n (%)		3 (6.1%)
Size of metastasis in SLN, n (%)	Macrometastasis (>2 mm)	39 (79.6%)
	Micrometastasis (0.2–2 mm)	10 (20.4%)
Findings after neck dissection, n (%)	Positive metastasis	16 (32.6%)
	Extranodal extension	9 (18.4%)

**Table 5:** Clinicopathological characteristics of cases with FN results after SLNB (n = 11).

False Negative	Age (Years)	Location	Tumour Size (mm)	DOI (mm)	PNI	Surgical Margin (mm)	SLN per Patient (n)	Contralateral SLN	Recurrence (Months)	Cervical Level of Recurrence	Salvage Therapy	Outcome
Case #1	62	Lateral tongue	25	3	No	2	3	No	10	II-III (ipsilateral)	RT/CT	DOD
Case #2	39	Lateral tongue	28	11	No	4	3	No	5	II-III-IV (contralateral)/M1	CT + IT	DOD
Case #3	63	Lateral tongue	26	5	No	0	1	Yes	1	II (ipsilateral)	RT/CT	DOD
Case #4	49	Lateral tongue	16	10	No	2.5	6	Yes	5	II-III-IV (ipsilateral)	RT/CT	DOD
Case #5	82	Lateral tongue	33	4.5	No	4	1	No	3	II (ipsilateral)	ND	NED
Case #6	83	Lateral tongue	34	6	No	5	2	No	5	I (ipsilateral)	RT	NED
Case #7	27	Lateral tongue	25	9.5	Yes	0	1	No	5	III (ipsilateral)/M1	CT + IT	DOD
Case #8	26	Ventral tongue	15	6	Yes	0	2	No	6	I (ipsilateral)	ND	NED
Case #9	63	Lateral tongue	22	13	No	5	4	Yes	5	II-III (contralateral)/M1	RT/CT	DOD
Case #10	61	Ventral tongue	17	4	No	0	4	Yes	3	I (ipsilateral)	RT/CT	NED
Case #11	61	Floor of mouth	25	6	No	5	3	No	4	II (ipsilateral)	ND	NED

Cervical levels I–V refer to standard neck nodal levels: Level I (submental/submandibular), level II (upper jugular), level III (mid-jugular), and level IV (lower jugular). M1: Distant metastasis. RT: Radiotherapy. CT: Chemotherapy. ND: Neck dissection. IT: Immunotherapy. DOD: Died of disease. NED: No evidence of disease.

**Table 6:** Comparison between FN and TN subgroups.

Variable		False Negative (n = 11)	True Negative (n = 160)	*p*-Value
Age (years)	median (IQR)	61 (44–63)	64 (57–72)	0.182
Tumour size (mm)	median (IQR)	25 (19.5–27)	15 (10–20)	0.0008
Depth of invasion (mm)	median (IQR)	6 (4.8–9.8)	3 (1.5–5.8)	0.0102
Perineural invasion	n (%)	2 (18.2%)	8 (5%)	0.089
Surgical margin (mm)	median (IQR)	2.5 (0–4.5)	4 (2–5)	0.313
SLN per patient	median (IQR)	3 (1.5–3.5)	3 (2–4)	0.186
Contralateral SLN	n (%)	4 (36.4%)	49 (30.6%)	0.741

## Data Availability

The data presented in this study are available by contacting the corresponding author upon reasonable request.

## Abbreviations

The following abbreviations are used in this manuscript:

DFS	Disease-free survival
DOI	Depth of invasion
DSS	Disease-specific survival
END	Elective neck dissection
ENE	Extranodal extension
FN	False negative
ND	Neck dissection
NPV	Negative predictive value
NSLN	Non-sentinel lymph node
OCSCC	Oral cavity squamous cell carcinoma
OS	Overall survival
PNI	Perineural invasion
SLN	Sentinel lymph node
SLNB	Sentinel lymph node biopsy
TN	True negative
TP	True positive
